# Predicting VO_2max_ in competitive cyclists: Is the FRIEND equation the optimal choice?

**DOI:** 10.3389/fphys.2023.987006

**Published:** 2023-02-06

**Authors:** Iva Jurov, Marta Cvijić, Janez Toplišek

**Affiliations:** ^1^ Clinical Institute of Occupational, Traffic and Sports Medicine, University Medical Centre Ljubljana, Ljubljana, Slovenia; ^2^ Department of Cardiology, University Medical Centre Ljubljana, Ljubljana, Slovenia

**Keywords:** maximal oxygen consumption, performance, FRIEND equation, indirect calorimetry, endurance capacity

## Abstract

Predicting VO_2max_ in athletes is vital for determining endurance capacity, for performance monitoring, in clinical diagnostic procedures, and for disease management. This study aimed to assess the most suitable equation for predicting VO_2max_ in competitive cyclists. Competitive cyclists (496 males, 84 females, Caucasian, 580 total) were included in the study from 1 January 2014 to 31 December 2019. Only subjects who were actively participating in national or international competitions and who were registered competitive cyclists and part of cycling teams at the time of the measurements were included. Subjects performed an incremental test on a cycle ergometer, and VO_2max_ was measured as indicated by a plateau in VO_2_. In addition, four prediction equations (the FRIEND, Storer, Fairbarn, and Jones) were used to estimate VO_2max_. The predicted VO_2max_ using the FRIEND equation was in good agreement with the measured VO_2max_ in male and female athletes. This was reflected by a high correlation with *r* = 0.684 for men and *r* = 0.897 for women (*p* = 0.000), with ICC = 0.568 (95% CI 0.184, 0.752) for men and ICC = 0.881 (95% CI 0.813, 0.923) for women. Total error was 1.56 and 1.48 ml/min/kg and a minimal bias of−3.6 and −1.1 ml/min/kg (men and women, respectively). Using other equations resulted in a slight decline in agreement with the measured standard. The FRIEND equation predicted VO_2max_ accurately with small total error, small prediction errors, and with the smallest constant error in our study cohort, indicating the potential value of using FRIEND equation also in competitive cyclists. This equation proved to have the highest accuracy both in male and female cyclists.

## Introduction

Cardiopulmonary exercise testing is used to define the functional capacity and prognosis in heart and lung disease patients (Palange et al., 2018). In addition to health measures, it is also used to assess performance abilities in athletes (Balady et al., 2010). Indirect calorimetry in exercise testing is considered the gold standard to detect maximal oxygen uptake (VO_2max_), but it requires a skilled technician and the use of standardized exercise treadmill protocols or cycle ergometry (Balady et al., 2010). Comparing measured values of VO_2max_ of an athlete to predicted VO_2max_ values of normally active people can lead to misdiagnosis. Since their VO_2max_ is superior, a decline in VO_2max_ could be overlooked if it is compared to non-athletic population prediction values. This could lead to a failure to detect overtraining. Accurately predicted VO_2max_ values are needed to determine whether measured VO_2max_ in an athlete is suboptimal, which can lead to further investigation of the cause.

If indirect calorimetry is not available, different predictive equations are used in evaluating functional capacity in patients and athletes. The generally used equation is the American College of Sports Medicine (ACSM) equation (Glass et al., 2007); however, it is based on VO_2max_ measurements at a submaximal load on relatively small numbers of non-athletic healthy young adults. In addition to some established equations widely used in the past (Jones et al., 1985; Wasserman et al., 1987; Arstila et al., 1990; Storer et al., 1990; Fairbarn et al., 1994), a new FRIEND (The Fitness Registry and the Importance of Exercise National Database) equation was described to have a good prognostic value in heart failure (Kokkinos et al., 2020), coronary artery disease patients (Jang et al., 2020), and in healthy adults (Myers et al., 2017; Kokkinos et al., 2018).

In healthy trained adults, different equations have already been compared to establish the most accurate one for predicting VO_2max_ (Malek et al., 2004), while data in competitive athletes are limited. The most accurate equation for healthy trained adults was the Storer equation (Storer et al., 1990; Malek et al., 2004). Since competitive athletes do not exhibit comparable average VO_2max_ values as trained adults (Balady et al., 2010), this study aimed to assess the most suitable equation for predicting VO_2max_ in competitive cyclists. In addition, we wanted to assess if gender affects the accuracy of the prediction equation used.

## Materials and methods

### Design

Cardiopulmonary exercise testing with indirect calorimetry was used to determine the VO_2max_ in subjects. We used a cross-validation design as per Malek et al., (2004) to determine which of the equations can estimate the measured VO_2max_ with best precision (Table 1). The cross-validation analyses were based on measuring VO_2max_ and comparing it to predicted VO_2max_ by calculating the constant error (CE, which is the mean difference for actual VO_2max_-predicted VO_2max_), Pearson’s product–moment correlation (r), standard error of estimate (SEE), and total error (TE) (Table 1). The equations compared to estimate the measured VO_2max_ were selected based on findings using traditionally known equations on aerobically trained men and women (Malek et al., 2004) (Table 2). In addition, the new FRIEND equation was also included in the analysis. Institutional ethical committee approved performing this research.

**TABLE 1:** Four equations were compared in the cross-validation design: Jones (Jones et al., 1985), Fairbarn (Fairbarn et al., 1994), Storer (Storer et al., 1990), and FRIEND equation (Kokkinos et al., 2018).

Jones	Male	${VO}_{2\max}\;\left(l/\min\right)=\left(0.046*BH\right)-\left(0.021*age\right)-4.31$
	Female	${VO}_{2\max}\;\left(l/\min\right)=\left(0.046*BH\right)-\left(0.021*age\right)-4.93$
Fairbarn	Male	${VO}_{2\max}\;\left(l/\min\right)=\left(0.023*BH\right)+\left(0.0117*BW\right)-\left(0.031*age\right)-0.332$
	Female	${VO}_{2\max}\;\left(l/\min\right)=\left(0.0158*BH\right)+\left(0.00899*BW\right)-\left(0.027*age\right)+0.207$
Storer	Male	${VO}_{2\max}\;(ml/kg)/\min\;)=\left(10.51*PO\;\left(watt\right)\right)+\left(6.35*BW\right)+\left(10.49*age\right)+519.3$
	Female	${VO}_{2\max}\;(ml/kg)/\min\;)=\left(9.39*PO\;\left(watt\right)\right)+\left(7.70*BW\right)+\left(5.88*age\right)+136.7$
Friends	Male	${VO}_{2\max}\;\left(ml/kg/\min\right)=1.76*\left(PO\;\left(watt\right)*6.12/kg\;BW\right)+3.5$
	Female	${VO}_{2\max}\;\left(ml/kg/\min\right)=1.65*\left(PO\;\left(watt\right)*6.12/kg\;BW\right)+3.5$

VO_2max_, maximal oxygen consumption; PO, maximal power output; BW, body weight; BH, body height.

**TABLE 2:** Prediction models used for cross-validation in this study.

				Male cyclists		Female cyclists	
	Analyzed cohort	Protocol	Mean age	N	Mean VO_2max_	N	Mean VO_2max_
FRIEND	Excluded if subjects were diagnosed with (a) a history of cancer (any kind); (b) cardio-vascular disease; (c) chronic obstructive pulmonary disease; (d) chronic kidney disease; and (e) peripheral artery disease. Also excluded were those whose exercise tests were terminated for abnormal clinical findings and/or before achieving voluntary maximal effort (peak respiratory exchange ratio <1.0) and those less than 18 years of age	Not determined	35.9 ± 12.1	3,378	42.43 ± 9.57 mL/min/kg	1,722	23.25 ± 10.01 mL/min/kg
Storer	Inclusion criteria: sedators, non-smokers, and apparently healthy adults	Start at 0 W + 15 W/min	Ages 20–70, evenly distributed	115	2773.5 ± 603.3 mL/min	114	1612.1 ± 393.8 mL/min
Fairbarn	Exclusion criteria: athletes, use of any medication that could interfere with exercise performance and/or heart rate response (e.g., digoxin, 8-adrenergic blocking drugs, sympathomimetics), abnormal resting ECG, or baseline spirometry findings	Start at 16 or 32 W + 16 W/min	Ages 20–80, evenly distributed	111	51.7 ± 11.4 mL/min/kg for age 20–29; not reported for the whole sample	120	43.9 ± 9.6 mL/min/kg for age 20–29; not reported for the whole sample
Jones	Exclusion criteria: athletes and subjects with history of serious illness or any chronic disorders	Start at 16.3 W + 16.3 W/min	Ages 15–71, evenly distributed	50	Not reported	50	Not reported

### Subjects

A total of 580 competitive cyclists (496 men and 84 women, all Caucasian) were included in the study. Only subjects who were actively participating in national or international competitions and who were registered competitive cyclists and part of cycling teams at the time of the measurements were included. Data were gathered in five consecutive years (2014–2019) by the same personnel. Informed consent was obtained from all cyclists before starting the procedures.

### Procedures

The test subjects had to refrain from intense physical activity 24 hours prior to it. All incremental tests were performed on a cycle ergometer (Cyclus 2, Leipzig, Germany) with their own bike after a 15-min warm-up. Two protocols were used based on age and body mass. They are modified Conconi cycling tests: cyclists under 17 years of age or weighing less than 50 kg started the protocol at 60 Watts and increased 15 Watts every minute (the 60 + 15 W protocol), and cyclists above 17 years of age and weighing more than 50 kg started the protocol at 100 Watts and increased 20 Watts every minute (the 100 + 20 W protocol). The workload was constantly increased until volitional exhaustion, meaning that participants themselves declared when their absolute maximum was reached and the test was to be terminated. The test was also terminated if the cycling cadence dropped below 60. Heart rate (Polar V800, Polar Electro, Kempele, Finland), ventilatory, and gas data (measured with a V2 mask, Hans Rudolph, United States, of appropriate size) were collected during the incremental test with a metabolic cart (K5, Cosmed, Italy). Using breath-by-breath data, the VO_2max_ was determined as the average of the 5-s highest values during the last 30 s of the incremental test. All participants had to reach a plateau in VO_2max_ and RER ˃1.0 for their result to be recognized as maximal exertion and included in this analysis (Howley et al., 1995). The plateau was determined visually by experienced technicians. All measurements were performed in the physiological laboratory, with an ambient temperature of 21°C. The metabolic cart was calibrated prior to each of the measurements.

### Statistical analysis

For statistical analysis, SPSS version 25.0 (IBM SPSS Statistics, Chicago, Illinois, United States) was used. Descriptive statistics (average ±standard deviation) were used to represent the data. Four equations were compared: Jones, Fairbarn, Storer, and FRIEND equation. Correlations were calculated using the Pearson correlation coefficient (r). Total error (TE), a measure of a combination of random and systematic error, was calculated. Constant error (CE) was used to determine how much error should be expected if a prediction model was used instead of actual measurement. Finally, standard error of the estimate (SEE), measuring the accuracy of the predictions made by a regression model, was calculated (Supplementary File S1). Dependent *t*-test was used to compare the mean difference between the measured and predicted VO_2max_. Alpha was adjusted by the Bonferroni procedure. The Bland–Atman test was used for presenting results to evaluate the agreement among measured and predicted VO_2max_ values (Watson and Petrie, 2010; Odor et al., 2017). Intraclass correlation coefficient (ICC) was used to determine the interrater reliability and was calculated using an absolute agreement definition.

## Results

The characteristics of the cyclists included in the study are presented in Table 3.

**TABLE 3:** Characteristics of cyclists included in the study.

	Males (N = 496)		Females (N = 84)	
Teams	Thirteen competitive cycling teams		Nine competitive cycling teams	
Protocols	Increments of 20 W/min (˃50 kg and age ≥17) or 15 W/min (≤50 kg)		Increments of 20 W/min (˃50 kg and age ≥17) or 15 W/min (≤50 kg)	

	Mean	Std. Deviation	Mean	Std. Deviation
Age (years)	17.14	2.72	20.18	5.59
Height (cm)	178.67	6.73	166.04	5.57
Body composition				
Body mass (kg)	66.72	7.58	57.45	6.46
Fat mass (%)	9.29	3.87	17.34	4.67
Fat free mass (%)	51.43	1.68	45.98	2.8
Maximal incremental test				
Maximal power output (W)	393.74	56.48	296.05	45.05
Maximal power output (W/kg)	5.90	0.56	5.19	0.79
Maximal heart rate measured (beats/min)	198.46	7.90	194.24	7.85
Maximal HR predicted (beats/min)	202.86	2.72	199.82	5.59
Maximal oxygen consumption (mL/min/kg)	63.43	5.49	54.82	7.02

The predicted VO_2max_ varied significantly between the four equations used, and mean values were different from measured mean by + 5.8% to −11,6% in male cyclists and by + 0.5% to −27% in female cyclists. However, we found that the FRIEND equation, when compared with the reference measured VO_2max_, was the most accurate for predicting VO_2max_ both in men and women.

**FIGURE 1:**
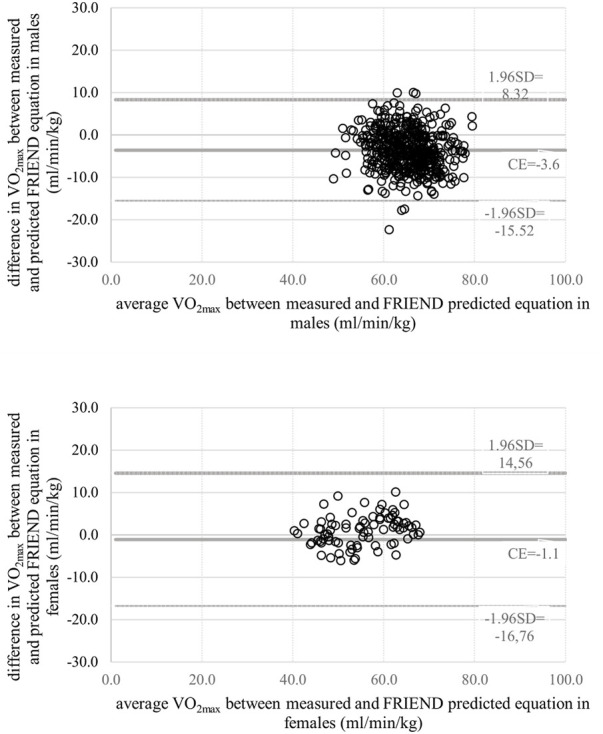
Bland–Altman plots comparing measured VO_2max_ and predicted VO_2max_ using the FRIEND equation for men and women.

In men, the predicted VO_2max_ using the FRIEND equation was in the most accurate agreement of all equations compared with the measured VO_2max_, reflected by a correlation with *r* = 0.684 (*p* = 0.000), ICC = 0.881 (95% CI 0.184, 0.752) and total error 1.56 ml/min/kg (Table 4), and a minimal bias of—3.6 ml/min/kg with the limit of agreement −15.52 and 8.32 ml/min/kg (Figure 1), while using other equations resulted in a slight decline in agreement with the measured standard (Table 4).

**TABLE 4:** Cross-validation of maximal oxygen uptake (VO_2max_) in male and female competitive cyclists.

									Bland–Altman analysis			Intraclass correlation		
										95% CI			95% CI	
Equation	Predicted VO2max (mL/min/kg) (mean)	SD	t	r	SEE (mL/min/kg)	SEE%	TE (mL/min/kg)	TE%	CE	Lower	Upper	ICC	Lower	Upper
Male cyclists (N = 496)														
FRIEND	67.08	6.08	−17.551	0.684*	4.013	6%	1.56	2.46%	−3.6	−15.52	8.32	0.568**	0.184	0.752
Storer	73.58	5.78	−50.156	0.682*	4.023	6.35%	5.53	8.72%	−10.1	−21.43	1.23	0.260**	−0.073	0.595
Fairbarn	60.89	5.12	7.350	−0.049	5.493	8.66%	2.94	4.64%	2.5	−7.54	12.54	−0.044	−0.123	0.037
Jones	53.52	4.08	31.698	−0.036	5.496	8.67%	6.58	10.38%	9.9	1.9	17.9	−0.011	−0.044	0.026
Female cyclists (N = 84)														
FRIEND	55.92	7.99	−2.847	0.897*	3.126	5.70%	1.48	2.70%	−1.1	−16.76	14.56	0.881**	0.813	0.923
Storer	56.88	7.20	−5.700	0.892*	3.196	5.83%	1.65	3.01%	−2.1	−16.22	12.02	0.856**	0.666	0.927
Fairbarn	49.30	5.76	6.007	0.142	6.988	12.74%	10.96	20.00%	5.5	−5.79	16.79	0.103	−0.066	0.279
Jones	40.05	5.04	16.325	0.083	7.035	12.83%	31.21	56.95%	14.8	4.92	24.68	0.020	−0.039	0.1

*Correlation is significant at the 0.01 level (2-tailed).

**ICC is significant at the 0.01 level.

*Alpha adjusted by Bonferroni procedure (P 0.05/4 = 0.0125).

(r, Pearson’s correlation coefficient; SEE, standard error of the estimate; TE, total error; CE, constant error; CI, confidence interval; ICC, intraclass correlation coefficient).

In women, we observed a wider range in predicted VO_2max_ values than in men (40.05–55.92 ml/min/kg). The predicted VO_2max_ using the FRIEND equation was in very good agreement with the measured VO_2max_, having a high correlation with *r* = 0.897 (*p* = 0.000), ICC = 0.881 (95% CI 0.813, 0.923) and a total error of 1.48 ml/min/kg (Table 4), and a minimal bias of -1.1 ml/min/kg with the limit of agreement -16.76 and 14.56 ml/min/kg (Figure 1), whereas in men, using other equations resulted in a slight decline in agreement with the measured standard (Table 4).

## Discussion

This study evaluated the accuracy of the equations for predicting VO_2max_ in a sample of 496 male and 84 female competitive cyclists. Subjects' characteristics confirmed that cyclists involved in the study were highly trained based on the measured VO_2max_ and relative power output (Faria et al., 2005a; Faria et al., 2005b) (Table 3).

Measuring total error could determine the difference between the measured VO_2max_ (true value) and predicted VO_2max_ (value derived from the equation) in each of the athletes. In the FRIEND equation, the total error was only 1.56 ml/min/kg, whereas in the Jones equation, it was 10.38 ml/min/kg in male cyclists. In the female counterpart, the differences are even greater, up to 31.21 ml/min/kg. VO_2_ measurements with an error of >10% are unacceptable (Palange et al., 2018), meaning that in male cyclists, the Jones equation is not appropriate. In female cyclists, only FRIEND (2.70%) and Storer (3.01%) equations could be used.

Looking for the strength of association between measured and predicted VO_2max_ in male cyclists, only the FRIEND and Storer equations showed a large positive strength of association to the measured VO_2max_. In female cyclists, the correlation was even higher than in male counterparts, whereas Jones and Fairbarn equations did not show any significant associations. The predicted VO_2max_ using the FRIEND equation was in very good agreement with the measured VO_2max,_ as supported by the highest ICC in both male and female athletes.

In the present study, the standard error of estimate was used to detect approximately how large the prediction errors (residuals) are for our data set. We found the smallest prediction errors in the FRIEND and Storer equations for both sexes. We can see that the variability was slightly lower in our female cyclists than in the male cyclists.

In both samples, male and female cyclists, we established that only the FRIEND and the Storer equations show small total error, a significant positive correlation with measured VO_2max,_ and small prediction errors. However, looking at constant error, in the male sample, the Storer equation resulted in large values, meaning that the calculated values deviate consistently from their true value to a much greater extent than in the FRIEND equation. We observed the same finding in female cyclists, but in this sample, the difference was smaller (−1.1 in FRIEND and −2.1 in Storer). In other words, the systematic error was the smallest when using the FRIEND equation for both males and females.

The FRIEND equation clearly seems superior to the Storer equation regarding both sexes, but differences are much smaller in the female sample. This might be due to a smaller sample size, but from the finding of the present study, we can suggest that both equations could be used. A reason why the FRIEND equation has a bigger correlation to measured values in female than in male cyclists could be due to the higher average age of our female samples—there is a smaller difference compared to average age in the FRIEND cohort. Another factor influencing performance is body fat, which can be extremely low in male cyclists and is associated with cycling performance (Jurov et al., 2020). Competitive female cyclists also have less body fat than normally active women (Martin et al., 2001), but their levels are not as extreme as in males, as was also the case in our study. Another advantage of the FRIEND equation is that unlike the Storer equation, age is not part of the calculation; only body weight and power output are.

In the present study, our sample was compared to prediction models (the Storer, Fairbarn, and Jones equations) that proved to be most accurate based on findings by Malek et al., (2004) that compared aerobically trained men and women. In addition, the new FRIEND equation was added to the comparison (Kokkinos et al., 2018). Prediction models made for specific populations may not be appropriate for populations with different characteristics (Zwiren et al., 1991; Kolkhorst and Dolaener, 1994; Malek et al., 2004; Myers et al., 2017; Kokkinos et al., 2018). Our two samples of 496 male and 84 female athletes are homogenous based on age, body composition, fitness, and activity level and are larger than the analyzed cohorts in prediction models (Table 2). The FRIEND registry is based on a larger cohort (3,378 men; mean age 35.9 ± 12.1 years), but considering the youngest age group, which is of interest when elite athletes are involved, the group is of similar size to our study sample (*n* = 505, age 20–29 years). This cohort is a few times bigger than the ones used in the Jones, Fairbarn, and Storer models, which could be one of the reasons for the better accuracy as shown in this study. Age in the Jones, Fairbarn, and Storer equation is higher than expected in competitive cyclists (Table 2). In the Fairbarn and Jones prediction models, age is also part of the equation. The older the person, the smaller the VO_2max_ (Table 1). We believe this could explain why the Fairbarn and Jones models overestimate VO_2max_, which results in a positive CE. The FRIEND and Storer models are not based on age, but on power output, which seems more appropriate. They underestimate VO_2max_, but absolute values of CE are smaller (Table 4; Table A1). In addition, the age span included is quite wide in all three mentioned models. All four models are based on inclusion criteria that used participants who were adults, and the activity level was not specifically determined. However, the Jones and Fairbarn model excluded athletes, and the Storer model included only sedentary individuals (Table 2). Since the FRIEND model did not exclude subjects based on vigorous activity or participation in competition, this might be the reason for the highest accuracy for predicting VO_2max_ in competitive cyclists of the models compared in the present study.

In general, there is a lack of cardiopulmonary testing protocol standardization (Palange et al., 2018), so it is challenging to get large cohorts of subjects with the same protocol, even more so if a specific population is studied, like competitive cyclists. The FRIEND registry is based on data obtained from different laboratories, and a specific protocol was not defined as part of the inclusion process. This is a disadvantage of the FRIEND equation, as the type of protocol can influence the VO_2max_ value (Midgley et al., 2008). We believe that using the same protocol as in our sample could result in even better accuracy of the predicted equation. Regardless of the lack of protocol standardization in the FRIEND prediction model, the equation proved to be the most accurate in our study. We assume that the large sample size in the FRIEND model could be the most important factor that affects the accuracy of the FRIEND equation.

### Limitations

There are some limitations that should be considered. Although the male sample in this study is the biggest sample of male competitive cyclists used in common studies, the female sample size is smaller. There are fewer female competitive cyclists in general, and to gather more data, we believe different laboratories should combine their data. Still, to the best of our knowledge, this is the biggest sample of female competitive cyclists using the same protocol, measurement equipment, and data collection procedures. In addition, the mean age of the male cyclists included in this study was slightly under 18 years, and we used prediction equations based on adults.

## Conclusion

Accurate prediction of VO_2max_ is vital in sports medicine (Sartor et al., 2013). For practitioners with no access to indirect calorimetry, it is the only way of assessing oxygen uptake at maximal exercise tolerance (Palange et al., 2018). Equations for VO_2max_ are useful also in field testing, which are very common in sports medicine and where indirect calorimetry is not always possible. When VO_2max_ measurement is available, predicted VO_2max_ can help identify possible decline in maximal values due to health impairment, like in cases of heart and lung diseases (Frederix, 2014). Since competitive athletes have a higher VO_2max_ than normally active adults, inaccurate predicted values can lead a physician to underestimate the severity of measured VO_2max_ or fail to recognize it at all. We demonstrated that the FRIEND equation predicted VO_2max_ most accurately with small total error, small prediction errors, and with the smallest constant error in our study cohort, indicating the potential value of using the FRIEND equation also in competitive cyclists. This equation proved to have the highest accuracy both in male and female cyclists. Since endurance athletes (like cyclists, triathletes, long distance runners) have similar body composition and endurance capacity requirements (Millet et al., 2009; Santos et al., 2014), this model might be appropriate also in the wider group of athletes. Further research is required to support or challenge our findings, to determine whether this model can be utilized in endurance disciplines, and to establish if athletes of other modalities (power disciplines and esthetic sports) show any dissimilarities.

## Data Availability

The raw data supporting the conclusions of this article will be made available by the authors, without undue reservation.
