# Supplementary material for: Predicting VO2max in competitive cyclists: Is the FRIEND equation the optimal choice?
Source: Front Physiol. 2023 Feb 6;14:987006. doi: 10.3389/fphys.2023.987006 (PMC9939680; doi:10.3389/fphys.2023.987006)
Supplement: Supplementary file 1 [file DataSheet1.docx]

Supplementary file 1

Table 5: Calculation of total error, constant error and standard error of the estimate used for cross-validation analysis

| total error (TE) | $\boldsymbol{TE=}\sqrt{\sum\left( {\boldsymbol{measured V}\boldsymbol{0}}_{\boldsymbol{2}\boldsymbol{max}}\boldsymbol{-predicted VO}_{\boldsymbol{2}\boldsymbol{max}} \right)^{\boldsymbol{2}}\boldsymbol{/n}}$ |
| --- | --- |
| constant error (CE) | $CE=\sum(measured {VO}_{2max}- predicted {VO}_{2max})/n$ |
| standard error of the estimate (SEE) | $SEE={SD}_{y}\sqrt{{(1-r}^{2})}$ |
| %TE | $\%TE =\frac{TE}{mean of measured {VO}_{2max}}*100$ |

VO_2max_ = maximal oxygen consumption, SD = standard deviation
